# The Questions of Age and Maximum Efficiency

**Published:** 1914-11-14

**Authors:** 


### THE ROYAL ARMY MEDICAL CORPS AND THE WAR.

### The Questions of Age and Maximum Efficiency.

Very early after the commencement of the operations on the Continent of the British Expeditionary Force the younger officers of the Royal Army Medical Corps began to complain that the senior commands of the Medical Service were in the hands of men who were past their best days. The traditional jealousy of crabbed age on the part of youth may possibly have accounted for some of this feeling, and this factor must of course be discounted. Another point to bear in mind, on the other side ot the account, is the better quality of officer which the R.A.M.C. has been attracting during the present century, owing to improved conditions of service; so that the junior officers of the present day do certainly reach a higher average level of efficiency than those of earlier years, now of senior standing. Be that as it may, it would almost seem as if there is some justification for the opinion held by the juniors, if one may judge by the changes in the Medical Service of one division, with which the present writer happens to be familiar; whether similar changes have occurred in other divisions he cannot say.

At the outset of the campaign the Assistant Director of Medical Services (A.D.M.S.) in this particular division was, as usual, a full colonel; under him were the three field ambulances of the division, each commanded by a lieutenant-colonel. The A.D.M.S. in question had a well-deserved reputation, founded on good work in bygone years; his health, however, was quite inadequate to the strain of the campaign, and he was soon invalided. His successor was another full colonel, and he, too, was shortly invalided. Finally, the post was filled by appointing the junior of the lieutenant-colonels commanding a field ambulance, an officer very much younger than either of the former. Each of the three field ambulances is now commanded by a major, and not in every case by one of very senior standing in that rank. It will therefore be seen that in this instance " youth will be served " is being proved a true adage. The fact is that, owing to the advanced age (compared with other branches of the Service) at which medical officers enter the Army, a full colonel in the Royal Army Medical Corps is often nearer sixty than fifty—of an age, in fact, corresponding with that of a lieutenant-general of the combatant arms. Although many men at this age are perfectly capable of directing the operations of war (witness a whole host of our own eminent soldiers), yet a great many are so no longer; and it is left to the practical test of active service to determine whether in individual cases the senior officers of the Royal Army Medical Corps are or are not equal to the demands which campaigning makes upon a man's physical and mental energies. It is significant in this connection that almost all the officers of the R.A.M.C. " mentioned in dispatches " by Sir John French are of the rank of major or lower.

During years of peace most Governments are disinclined, for so-called economical reasons, to retire men serving in branches of the Service which are non-combatant. It is held that if each such branch in the military department were kept at its maximum efficiency the Government would have to provide a much larger vote for it owing to the increased pensions, in addition to the full ordinary vote which must be provided. It has further been felt by Directors-General and others who have had to advise successive Governments that officers who are keenly efficient must be given adequate work to do, or they will grow discontented and leave the Service. So in practice it often happens, as it has undoubtedly happened in the case of the Royal Army Medical Corps, that retirements have been relatively few, and promotion has consequently been so slow as to cause much heartburning among the younger, and, indeed, among most of the abler members of the Corps who have kept themselves highly competent, and are so capable of doing the best work with the maximum of efficiency.

We have reason, from knowledge, justly to compliment the Medical Director-General of the Army and all responsible under him for the organisation and equipment of the hospital, ambulance and transport arrangements, whereby the present needs of the wounded in connection with our British Expeditionary Force have been provided for. There has been a care, a thoroughness, and a keen desire to secure the maximum efficiency in connection with all these arrangements. In France especially those responsible for the provision of facilities for the treatment of the wounded deserve, and should receive, the heartfelt thanks of the British Government and people. The facts that (1) Director-General Sir Alfred Keogh has actively participated in the provision and organisation of many of the arrangements for the wounded in France with the system pursued there, and (2) that at home the organisation under the control of the War Office in connection with the R.A.M.C. has proved in practice to be so commendably efficient, entitle the public to have confidence that the present arrangements are adequate and complete. Further, those responsible are fully alive to the continuous difficulties which have to be faced in the special circumstances, and judged by the work they have already accomplished, they may be trusted to prove equal to meet any emergency which may arise.

Of course, criticism has arisen and letters have been written to the Press by members of the profession and others who claim that the care of the wounded needs immediate supplementation, and that the staffs and equipment of the base hospitals in France should be strengthened by the dispatch from England of more hospital surgeons of experience. We have taken some trouble to ascertain the facts in this connection. We do not hesitate to say that the policy pursued by those in authority in this regard, and the choice they have made of eminent physicians and surgeons to proceed to the Front leave nothing to be desired, if the best interests of the patients and the efficiency of the hospital equipment are, as they must be, the first consideration. Sir Victor Horsley, if we may judge from a letter in the *Lancet* of the 7th inst., seems to have been unfortunate or exceptional in the information he has received about the condition in which the wounded have arrived in this country, as we have had the warmest expressions of appreciation from those who have received and have had to do with the treatment of the wounded, that the arrangements made for surgical attention during transport have been uniquely good. These arrangements have constituted a new feature for the better in the circumstances attending the removal of the wounded from the battlefield to the hospitals in this country. We have no hesitation, on the facts supplied to us, in declaring that the public may have the completest confidence in the efficiency of the ambulance and transport organisation for which the War Office and Sir Alfred Keogh have been responsible.

Reverting to the complaints amongst the younger officers of the R.A.M.C., in view of the fact that the war is expected to continue for some three years at least, we think that the War Office will be well advised at once to consider the whole of the questions raised. It would be wisdom to embrace the opportunity of the present state of war to encourage the retirement of many of the older men. This would secure that the whole of the *personnel* of the R.A.M.C. should attain the maximum of efficiency with the minimum of delay. Any extra expenditure entailed could properly be treated as a war item, and the additional cost would be more than repaid by the benefits likely to accrue to the Service from the adoption of this suggestion.

